# A Data‐Driven Psychiatric Disorder Subtype Defined by Significant Enlarged Ventricles and Cognitive Impairment: A Replication Study

**DOI:** 10.1002/npr2.70078

**Published:** 2026-01-14

**Authors:** Toshiaki Onitsuka, Yuka Yasuda, Satsuki Ito, Junya Matsumoto, Naohiro Okada, Maeri Yamamoto, Kazutaka Ohi, Tsutomu Takahashi, Manabu Kubota, Naoki Hashimoto, Harumasa Takano, Naomi Hasegawa, Go Okada, Reiji Yoshimura, Shin Nakagawa, Hidenori Yamasue, Shusuke Numata, Ryota Hashimoto

**Affiliations:** ^1^ NHO Sakakibara National Hospital Tsu Mie Japan; ^2^ Department of Neuropsychiatry, Graduate School of Medical Sciences Kyushu University Fukuoka Japan; ^3^ Life Grow Brilliant Mental Clinic, Medical Corporation Foster Osaka Osaka Japan; ^4^ Department of Pathology of Mental Diseases National Institute of Mental Health, National Center of Neurology and Psychiatry Tokyo Japan; ^5^ Department of Neuropsychiatry, Graduate School of Medicine The University of Tokyo Tokyo Japan; ^6^ International Research Center for Neurointelligence (WPI‐IRCN), The University of Tokyo Institutes for Advanced Study (UTIAS) The University of Tokyo Tokyo Japan; ^7^ Department of Psychiatry Nagoya University Graduate School of Medicine Nagoya Aichi Japan; ^8^ Department of Psychiatry Gifu University Graduate School of Medicine Gifu Gifu Japan; ^9^ Department of General Internal Medicine Kanazawa Medical University Uchinada Ishikawa Japan; ^10^ Department of Neuropsychiatry University of Toyama Graduate School of Medicine and Pharmaceutical Sciences Toyama Japan; ^11^ Research Center for Idling Brain Science University of Toyama Toyama Japan; ^12^ Department of Psychiatry, Graduate School of Medicine Kyoto University Kyoto Japan; ^13^ Department of Psychiatry Hokkaido University Graduate School of Medicine Sapporo Hokkaido Japan; ^14^ Department of Clinical Neuroimaging Integrative Brain Imaging Center, National Center of Neurology and Psychiatry Tokyo Japan; ^15^ Department of Psychiatry and Neurosciences, Graduate School of Biomedical and Health Sciences Hiroshima University Hiroshima Japan; ^16^ Department of Psychiatry University of Occupational and Environmental Health Fukuoka Japan; ^17^ Division of Neuropsychiatry, Department of Neuroscience Yamaguchi University Graduate School of Medicine Yamaguchi Japan; ^18^ Department of Psychiatry Hamamatsu University School of Medicine Shizuoka Japan; ^19^ Department of Psychiatry, Graduate School of Biomedical Science Tokushima University Tokushima Japan

**Keywords:** biologically homogeneous subgroup, cognitive impairment, enlarged ventricles, EVCI, schizophrenia

## Abstract

We previously performed data‐driven classification based on large‐scale neuroimaging datasets to identify a potential novel subtype of psychiatric disorders characterized by the pronounced enlarged ventricles and cognitive impairment (EVCI) in one cohort. However, these findings have yet to be validated across independent cohorts. Herein, we investigated the availability of cognitive data in other cohorts in the Cognitive Genetics Collaborative Research Organization (COCORO) project to assess the presence of cognitive impairment (CI). Subsequently, we compared the prevalence of CI, lateral ventricular volume, and demographic and clinical characteristics between individuals with EVCI and those with EV but without CI (EV‐nonCI). Cognitive data were available for 27 individuals with EVCI from the other 8 cohorts. Among the 27 individuals in the EV group, 5 (18.5%) exhibited EVCI, all of whom were diagnosed with schizophrenia. Three individuals (60%) in the EVCI group were male. Compared with the EV‐non‐CI group, the EVCI group presented significantly lower current intellectual quotient (IQ) scores (*p* = 5.8 × 10^−4^), but there was no significant difference in premorbid IQ (*p* = 0.56). Furthermore, there was no significant difference in the *Z* scores for lateral ventricular volume between the groups (*p* = 0.26). This study validated the existence of the EVCI subgroup in independent cohorts, thus supporting our previous findings.

## Abstract 2

This multicenter study replicated a biologically homogeneous psychiatric subtype characterized by enlarged lateral ventricles and cognitive impairment (EVCI). Among 27 individuals with enlarged ventricles, five (18.5%) exhibited EVCI—all diagnosed with schizophrenia and 60% male. These findings validated EVCI across independent cohorts, supporting its potential diagnostic relevance.

## Graphical abstract

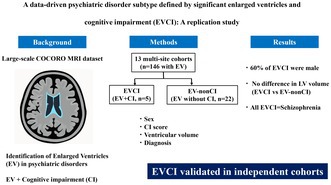

## 1 Introduction

An enlarged ventricle (EV) is a structural brain alteration that is observed in various psychiatric disorders. The presence of EV in patients with schizophrenia was documented by Johnstone et al. [1], and subsequent neuroimaging studies have consistently demonstrated a significant increase in lateral ventricular volume among individuals with schizophrenia compared with healthy controls (HC) [2]. However, EV may not be specific to schizophrenia, since this alteration has also been reported in other psychiatric disorders, such as bipolar disorder and major depressive disorder [3, 4].

Cognitive impairment (CI) is another well established hallmark of schizophrenia, as extensively documented in the literature [5]. In our previous study [6], we performed data‐driven classification based on large‐scale neuroimaging datasets to identify a novel subtype of psychiatric disorders characterized by pronounced EV and CI (i.e., EVCI). Although the diagnosis of psychiatric disorders predominantly depends on clinical symptomatology, incorporating magnetic resonance imaging (MRI)‐based evaluations of lateral ventricular volume alongside cognitive assessments could facilitate the development of a more objective and biologically grounded diagnostic framework.

In our previous investigation [6], we analyzed MRI data from 5602 subjects across 14 cohorts, and we identified 186 individuals with EV. CI was assessed using the Wechsler Adult Intelligence Scale‐III [7] (WAIS‐III) and the Japanese Adult Reading Test (JART) [8, 9]. Specifically, cognitive data were available for 33 out of 40 EV individuals in the cohort from Osaka University; 9 individuals (27.3%) exhibited EVCI, and 8 (88.9%) of the 9 EVCI individuals had a diagnosis of schizophrenia [6].

In the present study, we aimed to expand upon these findings by incorporating more supplemental cognitive function data from additional cases. We examined background characteristics, including cognitive function, in this EV sample.

## 2 Methods

The study was conducted in accordance with the principles outlined in the Declaration of Helsinki and received approval from the Ethics Review Board of the National Center of Neurology and Psychiatry (approval number B2023‐080) as well as local ethics committees. Written informed consent was obtained from all participants prior to study enrollment.

Regarding the MRI findings, the left and right lateral ventricular volumes were computed in our previous study [10], and the detailed methodology for their calculation was thoroughly described. These volumes were normalized according to the HC distribution, and linear regression models were constructed with adjustments for sex, age, and intracranial volume [11]. Individual *z* scores for the left and right lateral ventricular volumes were calculated for each subject. EV was operationally defined as an average *z* score greater than three for both lateral ventricle volumes. We identified 186 EV individuals as reported in a previous study. Since 40 EV individuals in the cohort from Osaka University had already been examined previously, we investigated the availability of cognitive assessments for the remaining 146 individuals with EV across 13 cohorts of the Cognitive Genetics Collaborative Research Organization (COCORO) project [12]. This EV sample consisted of HC (*n* = 23) as well as individuals diagnosed with schizophrenia (*n* = 86), bipolar disorder (*n* = 11), major depressive disorder (*n* = 24), and autism spectrum disorder (*n* = 2).

CI was assessed via the WAIS‐III [7], the WAIS‐IV [13], the simplified version of the WAIS [14, 15], and the JART [8, 9]. CI was quantified as the difference between the current intellectual quotient (IQ) and the estimated premorbid IQ [16, 17, 18]. Since the Japanese simplified version of the WAIS‐III and WAIS‐IV has been shown to provide adequate estimates of full‐scale IQ [14, 15], the current IQ was assessed using either the full version or the simplified version of the WAIS [14, 15], whereas premorbid IQ was estimated using the JART [8, 9]. The severity of CI was classified as follows: severe impairment (≥ 30‐point decrease), moderate impairment (20‐ to 30‐point decrease), mild impairment (15‐ to 20‐point decrease), borderline impairment (10‐ to 15‐point decrease), and within the normal range (< 10‐point change) [16, 17, 18]. Consistent with our previous study, individuals with EV who exhibited moderate or severe CI were considered to have EVCI [6].

SPSS version 28.0 (IBM, Tokyo, Japan) was used for statistical analysis. Categorical data were compared using the chi‐square test, and continuous data were compared using the Mann–Whitney *U* test.

## 3 Results

Cognitive function data were available in 8 out of the 13 cohorts. Furthermore, cognitive data were available for 27 of the 146 individuals with EV. Table 1 shows the biological characteristics of the patients. A total of 18.5% (*n* = 5) were classified as having EVCI. Three of the 5 (60%) individuals with EVCI were male.

**TABLE 1:** Diagnostic and biological characteristics of patients with enlarged ventricles and cognitive impairment.

	EVCI	EV‐non‐CI	*U* or *χ*	*p*
*N* (%)	5 (18.5%)	22 (81.5%)		
Female (%)	2 (40.0%)	5 (22.7%)	0.6	0.43^a^
Age, mean (SD)	52.4 (6.0)	44.7 (18.1)	39.5	0.34^b^
Years of education, mean (SD)	13.0 (2.6)	14.4 (2.2)	69.5	0.28^b^
Cognitive impairment score, mean (SD)	−23.4 (4.4)	0.9 (11.4)	105	**3.0 × 10** ^**−5**^ ^b^
Current IQ, mean (SD)	75.8 (4.5)	100.4 (15.5)	95.5	**5.8 × 10** ^**−4**^ ^b^
Premorbid IQ, mean (SD)	99.2 (3.6)	101.2 (11.2)	65	0.56^b^
*Z* score of ventricle volume, mean (SD)	4.2 (2.1)	4.3 (1.5)	73	0.26^b^
Psychiatric disorder classification: SZ/BP/MDD (% of SZ)	5/0/0 (100%)	13/4/5 (59%)	3.1	0.22^a^

*Note:* Diagnostic and biological features of subjects with enlarged ventricles and cognitive impairment (EVCI) and enlarged ventricles without cognitive impairment (EV‐non‐CI) are shown. Enlarged ventricles were defined as average *z* score of left and right lateral ventricle volumes greater than three. Cognitive impairment was defined as a decline of 20 points or more on the cognitive impairment score, which was defined as subtracting the premorbid IQ (Japanese Adult Reading Test: JART) from the current IQ (Wechsler Adult Intelligent Scale: WAIS). Statistically significant *p* values are bolded.

Abbreviations: BP, bipolar disorder; MDD, major depressive disorder; *N*, number of subjects; SD, standard deviation; SZ, schizophrenia.

^a^ Categorical data were compared using the chi‐square test.

^b^ Continuous data were compared using the Mann–Whitney test.

Because the grouping was inherently determined by the presence or absence of cognitive impairment, a significant difference between the two groups was inevitable; nevertheless, the results were as follows: the CI score was −23.4 ± 4.4 in the EVCI group and 0.9 ± 11.4 in the EV‐non‐CI group; this difference was statistically significant (*p* = 3.0 × 10^−5^). The current IQ was 75.8 ± 4.5 in the EVCI group and 100.4 ± 15.5 in the EV‐non‐CI group; this difference was statistically significant (*p* = 5.8 × 10^−4^). There were no significant differences in premorbid IQ between the two groups (99.2 ± 3.6 for EVCI; 101.2 ± 11.2 for EV‐non‐CI, *p* = 0.56). The *Z* scores for lateral ventricular volume did not significantly differ between the two groups (4.2 ± 2.1 for EVCI; 4.3 ± 1.5 for EV‐non‐CI, *p* = 0.26). These findings are consistent with the trends observed in our previous study (see Table S1, which is a modified version of table 5 in the previous paper [6]).

Notably, in the present study, all individuals classified as having EVCI had a diagnosis of schizophrenia, suggesting that most cases of EVCI occur in individuals with schizophrenia. Consistent with our previous study, the present study also revealed a higher prevalence of males in the EVCI group. No significant differences were observed between the EVCI group and the EV‐non‐CI group in terms of age, sex, or years of education.

## 4 Discussion

Although EV and CI are often observed in psychiatric disorders, there is no consensus regarding the diagnostic value of their combination. Yasuda et al. [6] performed a large‐scale data‐driven analysis and found that patients with EVCI constitute a distinct subgroup characterized by a high prevalence of schizophrenia. In the current multicenter study, among the 27 patients for whom both EV status and cognitive function data were available, all individuals diagnosed with EVCI also had schizophrenia, suggesting that most cases of EVCI occur in individuals with schizophrenia. Given that EVCI was identified in 27% of individuals with EV in a previous study, it is plausible that EVCI occurs in approximately 20% of individuals with EV. Additionally, the present study revealed a greater prevalence of males in the EVCI group. The CI scores and lateral ventricular volumes of the EVCI and EV‐non‐CI groups followed a similar pattern and provided additional support for our previous findings [6]. Although EVCI may represent an extreme phenotype that is only observed in a small subset of psychiatric disorders, its identification is particularly novel owing to its potential diagnostic value. The EVCI group identified in this study may constitute a biologically homogeneous subgroup that transcends conventional diagnostic classifications.

Previous studies have reported associations between the EVCI subgroup and both electroencephalography (EEG) abnormalities and specific genetic variants (e.g., 22q11.21 deletion, 7q11.23 duplication) [6]. Our recent report [19] further supports the notion that EVCI may represent a biologically homogeneous subgroup. In that report [19], patients with EVCI frequently exhibited treatment‐resistant schizophrenia with partial responsiveness to clozapine, ischemic changes in cerebral white matter, electroencephalographic abnormalities, and pathogenic copy number variations such as 22q11.21 deletion and 7q36.2 deletion. These findings suggest that EVCI may be associated with neurodevelopmental vulnerability and synaptic dysfunction, providing a potential biological framework for understanding the link between ventricular enlargement, cognitive impairment, and poor treatment response. In the future, integrating neuroimaging, cognitive, electrophysiological, and genetic data may further improve the precision of biological subtype classification in psychiatric disorders. Taken together, these findings suggest that EVCI represents a biologically homogeneous subgroup that transcends existing diagnostic categories, offering a novel perspective for the classification of psychiatric disorders.

This study has several limitations. First, the major limitation of this study is that cognitive function data were available for only 27 (18.5%) of the 146 participants with enlarged lateral ventricles. However, this missing data may be related to nonsystematic missingness arising from methodological constraints. Second, it should be noted that cognitive function data were available for only a subset of the EV group, resulting in an EVCI subgroup of only five participants and markedly limited statistical power. In future studies, large‐scale and prospective multi‐site collaborations that implement standardized assessments of cognitive function and brain imaging are needed to further validate the reproducibility of EVCI and to elucidate its neurobiological basis. Third, EEG findings and genetic information were not available in the current EVCI sample. In addition, since this study is cross‐sectional, further research is needed to determine whether EV is associated with pathophysiological progression or whether they represent a congenital trait. Longitudinal MRI studies analyzing structural brain changes and CI before and after disease onset will be essential to elucidate this issue. Finally, expanding sample sizes for psychiatric disorders beyond schizophrenia, such as major depressive disorder, bipolar disorder, and autism spectrum disorder, will be critical for validating current findings.

## 5 Conclusions

The present study validated the existence of the EVCI subgroup in independent cohorts. EVCI may be more frequently observed among males and is predominantly associated with a diagnosis of schizophrenia.

## Author Contributions

Toshiaki Onitsuka was involved in data collection and data analysis and wrote the first draft of the manuscript. Yuka Yasuda, Satsuki Ito, and Junya Matsumoto were involved in the data analysis and contributed to the interpretation of the data. Naohiro Okada, Maeri Yamamoto, Kazutaka Ohi, Tsutomu Takahashi, Manabu Kubota, Naoki Hashimoto, Harumasa Takano, Naomi Hasegawa, Go Okada, Reiji Yoshimura, Shin Nakagawa, Hidenori Yamasue, and Shusuke Numata were involved in the data analysis and contributed to the interpretation of the data. Ryota Hashimoto supervised the entire project, collected the data and was critically involved in the design, analysis, and interpretation of the data. All of the authors contributed to and approved the final manuscript.

## Funding

This research was supported by AMED Grant Numbers JP18dm0307002 (R.H.), JP21dk0307103 (R.H.), JP21wm0425012 (J.M. and R.H.), JP22tm0424222 (R.H.), JP24dk0307132 (R.H.), and JP24WM0625302 (T.T.); JSPS KAKENHI Grant Numbers JP19H05467: Grant‐in‐Aid for Specially Promoted Research (R.H.), JP20H03611: Scientific Research B (J.M., N.H., and R.H.), JP20K07945 and JP23K07001: Scientific Research C (J.M.), JP23H00395, JP25K10866 (T.O.): Scientific Research A (R.H.), and JP25K17172: Early‐Career Scientists (S.I.), JP24K10704 (T.T.); Intramural Research Grants for Neurological and Psychiatric Disorders of NCNP (Grants 3‐1, R.H.; 6‐1, R.H.; and 7‐5, J.M. and R.H.); and SENSHIN Medical Research Foundation (T.O.).

## Conflicts of Interest

The authors declare no conflicts of interest.

## Supporting information


**Table S1:** Diagnostic and biological features of individuals with enlarged ventricles and cognitive impairment (EVCI) in the previous report.

## Data Availability

The datasets generated and analyzed during the current study are not publicly available due to ethical and privacy restrictions. Data sharing is possible only within the framework of formal research collaborations approved by the corresponding institutions.
