# Supplementary material for: A Data‐Driven Psychiatric Disorder Subtype Defined by Significant Enlarged Ventricles and Cognitive Impairment: A Replication Study
Source: Neuropsychopharmacol Rep. 2026 Jan 14;46(1):e70078. doi: 10.1002/npr2.70078 (PMC12803960; doi:10.1002/npr2.70078)
Supplement: Supplementary file 1 — Table S1: Diagnostic and biological features of individuals with enlarged ventricles and cognitive impairment (EVCI) in the previous report. [file NPR2-46-e70078-s001.docx]

**Supplementary Table S1. Diagnostic and biological features of individuals with enlarged ventricles and cognitive impairment (EVCI) in the previous report**

|  | EVCI | EV-nonCI | *P* value |
| --- | --- | --- | --- |
| N (%) | 9 (27.3%) | 24 (72.7%) |  |
| Female (%) | 3 (33.3%) | 4 (16.7%) | 0.36^†^ |
| Age, mean (SD) | 31.6 (11.5) | 39.1 (19.1) | 0.49^‡^ |
| Years of education, mean (SD) | 13.1 (1.8) | 14.3(2.7) | 0.19^‡^ |
| Cognitive impairment score, mean (SD) | -27.1 (5.2) | -3.8 (10.3) | **5.2 × 10^-8‡^** |
| Current IQ, mean (SD) | 66.9 (10.5) | 102.9 (14.7) | **1.6 × 10^-6‡^** |
| Premorbid IQ, mean (SD) | 94.0 (13.2) | 106.7 (10.3) | **0.011^‡^** |
| Z-score of Ventricle volume, mean (SD) | 4.3 (1.1) | 4.3 (1.6) | 0.54^‡^ |
| Psychiatric disorder classification: SZ/BP/MDD/ASD/OM/HC (% of SZ) | 8/0/0/1/0/0 (88.9%) | 9/0/1/4/1/9 (37.5%) | **0.017^§^** |
| EEG abnormality (abnormal/total) (%) | 5/7 (71.4%) | 0/11 (0%) | **2.5 × 10^-3§^** |
| Rare CNV (N/total) (%) | 3/7 (42.9%) | 0/12 (0%) | **0.036^§^** |

Diagnostic and biological features of individuals with enlarged ventricles and cognitive impairment (EVCI) and those with enlarged ventricles without cognitive impairment (EV-nonCI) are shown. An enlarged ventricle was defined as an average z score greater than three for the left and right lateral ventricle volumes. Cognitive impairment was defined as a decrease of ≥20 points in the cognitive impairment score, which was calculated by subtracting the premorbid IQ (Japanese Adult Reading Test [JART]) from the current IQ (Wechsler Adult Intelligence Scale [WAIS]). N, number of subjects; SD, standard deviation; SZ, schizophrenia; BP, bipolar disorder; MDD, major depressive disorder; ASD, autism spectrum disorder; OM, other mental disorders (personality disorder, conversion disorder, attention-deficit/hyperactivity disorder, social anxiety disorder or psychotic disorder not otherwise specified); CNV, copy number variation. As there were missing data for EEG and copy number variation, available data (EEG: seven individuals in EV and CI and eleven individuals in EV-nonCI; CNV: seven individuals in EV and CI and twelve individuals in EV-nonCI) were analyzed. Categorical data were analyzed with either the chi-square test† or Fisher’s exact test§, and numerical data were analyzed with the Mann–Whitney test^‡^. Statistically significant associations (*P* value < 0.05) are highlighted in bold and underlined.
